# Quality of life and age following stroke

**DOI:** 10.18632/aging.101797

**Published:** 2019-01-28

**Authors:** Monique F. Kilkenny, Rohan Grimley, Natasha A. Lannin

**Affiliations:** 1 Stroke and Ageing Research, School of Clinical Sciences at Monash Health, Monash University, Victoria, Australia; 2 Stroke Division, Florey Institute of Neuroscience and Mental Health, University of Melbourne, Heidelberg, Victoria, Australia; 3 Sunshine Coast Clinical School, The University of Queensland, Birtinya, Queensland Australia; 4 School of Allied Health, La Trobe University, Bundoora, Victoria, Australia; 5 Alfred Health, Melbourne, Victoria, Australia; On behalf of the AuSCR Consortium

**Keywords:** stroke, quality of life, transient ischemic attack

Stroke is a major health problem with a significant impact on the health-related quality of life [1]. Registry data, such as that collected in the Australian Stroke Clinical Registry, provide information that can be used in clinical decision-making and monitor the quality of care and outcomes of those hospitalised for stroke [2,3]. Measures that assess health-related quality of life, such as the EuroQoL-5 dimension-3 level, reflect the importance of evaluating care and outcomes from the patients’ own perspective. The EuroQoL-5 dimension-3 level is a validated measure and includes a visual analogue scale where the patient rates their health status from 0 to 100 (0 worse than death and 100 best health) [4]. Five further questions summarise the patient’s levels of mobility, pain or discomfort, self-care, anxiety or depression, and usual activities at 90-180 days follow-up post-stroke. Collecting these data have been important to describe the burden of stroke and as such, are of interest to healthcare providers, researchers and policymakers alike.

In recent research published by our group, we reported that in patients who were admitted with stroke, those who required an interpreter were more likely to report a poorer health-related quality of life including more problems with self-care, pain, anxiety or depression and usual activities [5]. Beyond this finding, we also noted that patient and clinical characteristics differed between those who reported lower than median VAS score (<70) and those who reported above median VAS score (70+). Patients who reported below median VAS score were older (median age 77 years versus 72 years; p<0.001), more often female, had a documented history of a previous stroke, had experienced an in-hospital stroke and were less often able to walk at the time of admission or have had a transient ischemic attack as compared to those who reported above median VAS score (70+). This was despite these patients receiving similar or better quality of care.

Using the registry (AuSCR) data, Cadilhac et al. reported that the quality of care patients receive during their acute hospital stay can influence their long-term health-related quality of life [6]. Patients who were treated in a stroke unit reported a better health-related quality of life according to the VAS (coefficient, 21.34; 95% confidence interval, 15.50–27.18) than those patients not treated in a stroke unit. Thus, while age appears a key determinant of health-related quality of life post-stroke from our work, care on a stroke unit may reduce death and disability irrespective of age. Also using registry (AuSCR) data, Lannin et al. [7] confirmed that there were no differences between two age groups (<65 and 65 year or more) in terms of provision of care in a stroke unit. While care on a stroke unit appeared beneficial irrespective of age, consistent with other research on ageing and health-related quality of life post-stroke, Lannin et al. [7] did demonstrate a trend between increasing age with decreasing self-reported overall health status (85 median VAS score at 18-24 years to 69 median VAS score in the age group 75 year or more).

There remain many other factors that impact on health-related quality of life and are amenable to intervention. Implementation of interventions to address factors such as psychosocial issues. Thayabaranathan et. al. [8] used registry (AuSCR) data linked with administrative data to show that almost one in two patients at follow-up report some level of anxiety or depression after stroke. One of the strongest factors associated were a prior diagnosis of anxiety or depression. Unmet needs post stroke also remain a gap waiting to be filled. Andrew et al. [1] demonstrated that those who reported who had a reduced level of health-related quality of life post- stroke (based on the VAS score) reported a greater number of unmet needs a median of two years post-stroke.

Further research in older persons with stroke is required to understand the pre-morbid quality of life is the same, better or worse before their stroke. These data support the need to assess quality of life as it can be an indication of poor or successful ageing process. Interventions are required to improve the quality of life in older cohort of patients with stroke, especially patients who required an interpreter.

In conclusion, use of clinical registry data which includes data on health-related quality of life not only offers the opportunity to examine the “real life” interactions between disease states, clinical processes of care and meaningful outcomes for patients, but also form the foundation for translational research to simultaneously implement and demonstrate efficacy of these interventions.
